# The Child Life Challenges Scale (CLCS): Associations of a Single-Item Rating of Global Child Adversity with Children’s Total Life Stressors and Parents’ Childhood Adversity

**DOI:** 10.3390/children7040033

**Published:** 2020-04-10

**Authors:** Jillian S. Merrick, Madelyn H. Labella, Angela J. Narayan, Christopher D. Desjardins, Andrew J. Barnes, Ann S. Masten

**Affiliations:** 1 Department of Psychology, University of Denver, Denver, CO 80210, USA; Angela.Narayan@du.edu; 2 Department of Psychology, University of Delaware, Newark, DE 19716, USA; mlabella@psych.udel.edu; 3 Department of Mathematics and Statistics, St. Michael’s College, Colchester, VT 05439, USA; cddesjardins@gmail.com; 4 Department of Pediatrics, University of Minnesota, Minneapolis, MN 55455, USA; drbarnes@umn.edu; 5 Institute of Child Development, University of Minnesota, Minneapolis, MN 55455, USA; amasten@umn.edu

**Keywords:** childhood adversity, homelessness, measurement, psychological distress, stressful life events

## Abstract

Background: Although many existing measures tabulate specific risk factors to yield cumulative risk indices, there is a need for low-burden strategies to estimate general adversity exposure. Aims and Methods: This study introduces a brief, new measure of lifetime adversity, the Child Life Challenges Scale (CLCS), and examines its validity in a sample of parents and children residing in emergency housing. The CLCS comprises a single global item for rating cumulative life challenges utilizing either a paper-pencil scale or a sliding scale on a tablet. Parents are provided with anchor examples of mild and extreme challenges and asked to mark a location along the scale reflecting number and severity of challenges in their children’s lives to date. Study participants included 99 parents and their 3- to 6-year-old children. Results: CLCS scores were moderately associated with children’s parent-reported total life stressors, and these associations were robust to controls for parental history of adversity, parental distress, and family demographics. Control variables also did not moderate associations between CLCS scores and total life stressors, suggesting that the CLCS functions similarly across a range of sociodemographic risk. Paper-pencil and tablet versions showed similar convergent validity. Conclusion: The CLCS shows promise as an efficient measure for estimating children’s lifetime adversity with minimal parent or administrator burden.

## 1. Introduction

Young children exposed to adversity are at heightened risk for a range of negative outcomes across the lifespan [1]. Adverse experiences can include exposure to traumatic events, childhood maltreatment, family dysfunction, community violence, financial hardship, and other stressful life experiences and are known to pose risks to children’s healthy social-emotional development. For example, childhood maltreatment and exposure to family dysfunction are linked with mental and physical health problems in later childhood and in adolescence [2,3,4], and these associations often persist into adulthood [5,6,7]. Children exposed to early adversity tend to have worse social, emotional, and academic functioning at school entry [8] as well as increased rates of interpersonal difficulties and suicidal behavior throughout adolescence [9]. Adults with a childhood history of adversity report higher levels of unemployment and lower levels of educational attainment as well as lower overall life satisfaction and wellbeing [10,11,12]. Additionally, adverse experiences in childhood are associated with problems in romantic relationship functioning [13,14,15] and parenting behavior [16,17], contributing to patterns of intergenerational continuity in family adversity [18,19]. 

Such findings have motivated increased interest in measuring early adversity and its associated outcomes. Currently, many existing measures of adversity tabulate specific risk factors or life events to yield a cumulative risk index [20,21]. For instance, the Adverse Childhood Experiences (ACEs) scale uses this approach by asking about the presence or absence of 10 childhood adversities reflecting abuse, neglect, and family dysfunction. The number of adverse experiences endorsed are summed, yielding a total ACE score [5,6]. Various measures of stressful life events have been developed over the decades to provide similar cumulative estimates of risk and adversity, totaling events over different time intervals, including the past year or lifetime to date [22,23,24,25].

Adversity exposure in childhood often is assessed using adults’ retrospective self-report, as in the case of the ACEs scale. However, adverse experiences can also be assessed throughout childhood using parent- and/or self-reports. Indeed, early life event questionnaires were developed for parents to report on child life experiences, which was important for research that included younger children [26,27]. Trauma-focused researchers have also developed parent-report measures of traumatic life events [28,29]. Over time, measures have also been developed for older children and adolescents to self-report on their own life experiences [30,31,32]. 

Most of the questionnaires and checklists developed to assess negative life experiences in childhood rely on the cumulative risk approach, which has many strengths. This approach accurately reflects the co-occurrence of multiple risk factors, demonstrating that risk factors rarely occur on their own [20,21]. By tabulating discrete risk factors to form an overall index of risk, investigators were able to identify risk gradients, such that higher levels of risk or negative events were associated with worse outcomes in multiple areas of health, behavior, and development [33,34,35]. Furthermore, overall levels of risk often are better predictors of outcomes than any individual risk factor [36]. 

Despite these strengths, there are several shortcomings to the cumulative risk approach. Past reviews have identified multiple drawbacks, including difficulty selecting which risk factors should be included in the total score and the fact that intensity, chronicity, and duration of experiences are lost when simply dichotomizing whether an event occurred [20,21]. Such limitations have prompted the search for alternative strategies for measuring adversity. 

Additionally, although some participants tolerate adversity-related questionnaires well, evidence indicates that some participants experience questionnaires on stressful life events as aversive or evocative of negative emotions [37]. Measures enumerating specific adversities can be emotionally distressing for respondents, particularly if they are currently experiencing symptoms of depression or trauma or have experienced high levels of trauma exposure [38,39,40]. Trauma-specific questions may also be emotionally invasive, triggering, and taxing to complete for some participants [41,42,43]. In contrast, some studies have noted that participating in research that assesses exposure to traumatic events is a neutral to positive experience for individuals who have experienced or are currently experiencing stress or adversity [43,44,45,46,47]. Given this mixed body of evidence, participants’ willingness to respond to these measures is an important consideration in evaluating measures of adversity. Viable alternatives—brief but valid indices of adversity—are currently lacking. 

The sensitive nature of adversity-related questionnaires may also contribute to low rates of child adversity screening in clinical care settings. Although there are multiple settings where it may be both appropriate and beneficial to ask questions about children’s stressful life events and adversity exposure (e.g., physicians’ offices and home visits), this practice is not routine [48,49,50]. For instance, when pediatricians were questioned about their practices assessing children’s exposure to adversity in a primary care setting, 32% reported that they usually did not ask about any adverse experiences and only 2% reported that they routinely used a tool to screen for childhood adversity [51]. In one study, a majority of physicians (89%) noted that limited time to ask these questions was one of the major barriers to asking patients about their adversity histories, second only to limited time to counsel (92%) [50]. When residents and nurse practitioners were asked similar questions about their screening practices, they too reported low rates of adversity-related screening [52,53]. These professionals noted similar concerns and identified discomfort asking sensitive questions and worries about offending patients as additional barriers. Although past research has demonstrated that clinical screening for adversity is feasible, acceptable, and often supported by patients, parents, and providers [45,54,55,56,57], it is not common practice. This observation highlights the need for screening methods that directly address these concerns yet allow for children’s adversity to be easily assessed in clinical and primary care as well as in research settings. 

### The Present Study

**Figure 1:**
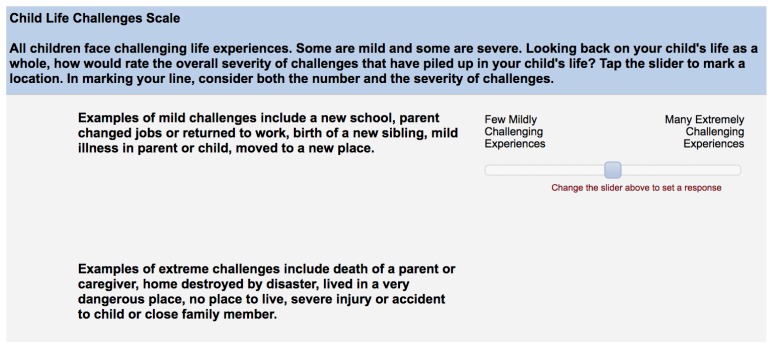
The Child Life Challenges Scale (tablet version).

**Figure 2:**
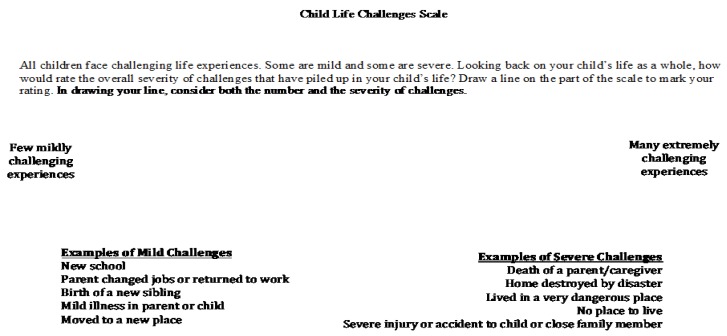
The Child Life Challenges Scale (paper-pencil version).

The current study introduces the Child Life Challenges Scale (CLCS), a brief, new parent-report measure of children’s lifetime adversity. The CLCS is a continuous scale developed to provide a quick estimate of children’s lifetime exposure to stressful and adverse experiences. Caregivers are provided with anchor examples of mild and extreme “challenges” (as the word “stress” or “adversity” can have markedly different meanings to different people) and asked to rate their children’s total experiences as a single mark (cumulative “life challenges”), using either a sliding scale administered on a tablet (Figure 1) or by marking a line on a paper-pencil scale (Figure 2). The CLCS is scored by measuring the distance of the marked line relative to the left endpoint (a score of “0” or “few mildly challenging experiences”) of the scale. In preliminary research by our group, the paper-pencil version of the CLCS demonstrated excellent test-retest reliability over a 1–2-week period (*r* = 0.80, *p* < 0.001) [58]. This study examines the measure’s validity in a sample of parents and children residing in emergency housing and examines its robustness against influences that might bias parental reports. 

The aims of the study were (1) to examine whether CLCS scores are an accurate reflection of the total life stressors that children have experienced as measured by a more traditional life events questionnaire; (2) to examine whether these associations were similar for the paper-pencil and tablet versions of the measure; (3) to examine whether total life stressors and CLCS scores were related after accounting for potential covariates that may influence the strength of their association, including parental history of adversity, parental distress, and family demographics; and (4) to explore whether these same parental adversity, parental distress, and demographic variables moderated the association between total life stressors and CLCS scores. Previous research has documented that parents’ own childhood adversity affects how they report their children’s experiences of adversity [59] and that adults’ inconsistencies in reporting their own adversity may be linked to current distress [60]. These studies raise the possibility that parental variables may moderate the relation between parents’ reports of children’s total life stressors and CLCS scores, with important implications for validity and interpretability of the CLCS. 

This study was grounded in the broad conceptual framework of developmental psychopathology [61], which emphasizes the importance of multiple experiences, negative or positive, in shaping development. More specifically, hypotheses were guided by the concept of cumulative risk, derived from evidence that risk factors for development—including both sociodemographic risk indicators and adverse life experiences—tend to co-occur, and when they do, greater risk to health or development is often observed than risk posed by any single risk factor [20,21]. Related theory suggests that cumulative risk indicators indirectly assess a history of multiple challenges over time that contribute to wear and tear on the body described by McEwen as allostatic load [62].

Regarding Aim 1, we hypothesized that the objective count of children’s parent-reported total life stressors from a traditional checklist would be significantly associated with children’s parent-reported CLCS scores. Regarding Aim 2, we hypothesized that tablet and paper-pencil CLCS scores would be highly correlated and would demonstrate a similar pattern to total reported life stressors. Regarding Aim 3, we hypothesized that CLCS scores would be related to total life stressors after accounting for these potential covariates. Aim 4 was exploratory; thus, we did not have specific expectations in regard to this aim.

## 2. Materials and Methods

### 2.1. Participants

Participants were recruited from a large, urban emergency shelter for homeless families, where the senior investigators have longstanding connections and space for research. Recruitment took place during the summers of 2016 and 2017. Families were considered eligible if they had been staying in the shelter for three consecutive days (to allow for acclimation), if children did not have severe developmental delays, and if caregivers and children spoke English well enough to participate in the assessment. Overall, 52% of all eligible families were recruited and the majority of families who did not participate were unable to be contacted or scheduled before they moved out of the shelter or before the study ended. Eligible participants were recruited at informational tables set up during mealtime and via fliers that were placed in the mailboxes of eligible families. This study was approved by the University of Minnesota Institutional Review Board (IRB) under two protocol names and numbers: “School Success in Motion: Reliability of School Readiness Measures” (2016 data collection; #1507S75122) and “School Success in Motion 2017: Parent and Child Well-Being in Families Experiencing Homelessness or High Mobility” (#STUDY00000358).

### 2.2. Procedure

The University of Minnesota’s IRB approved all study procedures. After providing informed consent, caregivers completed a series of questionnaires on family demographics, stress and adversity (including the CLCS), mental health, and wellbeing. For all participants, measures were read aloud to account for differences in reading level. 

Approximately one to two weeks after the initial session (*M* = 9.28 days), families were invited to return for a second follow-up session that also lasted approximately one hour; 87% of participants returned. During this session, they responded to similar measures asking about themselves and their children. Data collection was conducted over two sessions in order to gather retest reliability data on some of the instruments being collected, including the CLCS. Unless otherwise noted, the measures described in this paper were only collected at one of the two sessions. We note the session at which each instrument was collected in the Measures section below. Given the young age of the children participating as well as the number of instruments completed as a part of the larger study, we also conducted data collection over two sessions in order to be mindful of session length. Upon completion of each session, caregivers were compensated with an honorarium and children received a small toy. All participants received the same honorarium. 

### 2.3. Measures

**Child Life Challenges Checklist (CLCS):** All participants completed the CLCS, a single-item measure of global adversity in the child’s lifetime, during the initial session. Caregivers were provided with anchor examples of mild challenges (e.g., new school and parent changed jobs) and extreme challenges (e.g., death of a parent or caregiver and lived in a dangerous place) and were asked to note the point along a scale that represented their children’s total challenges, from 0 (few mildly challenging experiences) to 100 (many extremely challenging experiences). Instructions requested caregivers to consider both “the number and severity of challenges that had piled up in their child’s life” in marking their lines. The CLCS was administered to parents either in paper-pencil format or on a tablet, where they were instructed to tap along a sliding scale on the screen to mark their rating. The paper-pencil version of the measure was independently scored by two undergraduate research assistants, and all scoring discrepancies were examined and resolved by the first author. The tablet version of the measure was scored automatically using the survey platform on which data were collected. The tablet CLCS was administered to all participants during the first session. In the current sample, tablet CLCS scores ranged from 0 to 100 (*M* = 36.30, *SD* = 26.98). A subset of participants also completed the paper-pencil version of the CLCS and did so during the second session. They were asked to mark their rating on an approximately 20 cm line. Paper scores were computed by measuring the distance of the line (in cm) from the left anchor and were converted to match the tablet scoring from 0 to 100. For the paper-pencil version of the measure, scores ranged from 4.62 to 95.90 (*M* = 46.86, *SD* = 25.34). 

**Children’s Total Life Stressors:** An objective count of children’s total life stressors was measured using a version of the Lifetime Events Questionnaire (LTE-C) [63] that asked caregivers to report whether their child had ever experienced a list of specific negative events (e.g., a family member of the child ran away from home and one of the child’s parents lost his or her job). An index of lifetime adversity was computed by summing all endorsed events (total = 24), consistent with the cumulative risk approach [21] (*M* = 5.59, *Mdn* = 5.00, *SD* = 3.69, range = 0–16). The LTE-C was completed during the second session so that parents would not be primed by this measure before completing the CLCS.

**Parental Adverse Childhood Experiences (ACEs).** Parental childhood adversity was also assessed during the second session using the ACEs questionnaire [5]. This instrument includes 10 items that assess the presence or absence of adversities (i.e., physical, emotional, and sexual abuse; physical and emotional neglect; parental separation or divorce; and exposure to domestic violence, substance abuse, household mental illness, and incarceration) between birth and 18 years of age. Positively endorsed items were summed to yield a total ACE score (*M* = 4.89, *Mdn* = 5.00, *SD* = 3.02, range = 0–10). Parental ACEs were also examined dichotomously, as an ACE score of ≥4 is associated with multiplicative long-term health risk compared to an ACE score of three or fewer [64]. In the current sample, 50% of parents had ACE scores ≥ 4. 

**Parental Distress:** Parental distress was assessed using the Kessler Scale for Psychological Distress (K-6) [65]. The K-6 assesses nonspecific psychological distress (e.g., feeling that everything was an effort and feeling worthless) over the past month. Items are rated on a 0–4 scale, with lower scores indicating higher levels of distress. Total scores are computed by summing all items (*M* = 15.53, *SD* = 5.51, range = 0–24). According to the instrument developers, scores of 0–12 are considered above the threshold for psychological distress, whereas scores ≥ 13 are considered below the threshold for psychological distress. In the current sample, 25% of parents met this clinical threshold. Parental distress was assessed during the second session in summer of 2016 and during the first session in the summer of 2017. 

**Family Demographics:** Child age, child sex, and parental education were examined as demographic factors that may influence the relationship between objective count of parent-reported child stressful life events and CLCS scores. Descriptive statistics for child age and sex are reported below. There was a wide distribution in parental education, ranging from middle school through post-graduate education. Accordingly, education was dichotomized into two groups: less than a high school education (35%) and a high school degree or equivalent and greater (65%). All family demographics information was collected during the first session. 

### 2.4. Data Analytic Plan and Missing Data

To address Aim 1, bivariate correlations between the CLCS and children’s total life stressors were examined as were the bivariate associations between the CLCS and proposed continuous covariates. We also conducted independent samples t-tests to examine differences among CLCS scores and ordinal and dichotomous covariates and examined the correlations between the paper-pencil and tablet versions of this measure and between the paper-pencil version of this measure and children’s total life stressors. To address Aim 2, we examined the correlations between the paper-pencil version of the CLCS and children’s total life stressors. These analyses were conducted using a subset of the total sample (*n* = 34 participants) who had also been administered the paper-pencil version of this measure one to two weeks after completing the tablet version as a part of the larger study protocol in the summer of 2017. For Aim 3, which examined the association between children’s total life stressors and CLCS scores (dependent variable) when controlling for demographic variables, total parental distress, and total parental ACE, we compared two models (one containing just the covariates and one with the covariates and children’s total life stressors) and examined the change in R^2^. For Aim 4, which tested potential moderation of the association between CLCS scores and children’s total life stressors by each of the proposed covariates, interactions between parental distress, parental ACEs, and each demographic variable were examined in separate models. All regressions were examined for influential cases using Cook’s *d* ≥ 4/*n* [66,67].

For participants missing only one item on children’s total life stressors, parental ACEs, or parental distress, a total score was computed by summing all remaining items. When participants were missing two or more items on these instruments, total scores were considered missing. Using this guideline, the proportion of missing data ranged from 0% (on some demographic variables) to 18% (on ACEs). Data were missing for 11% of CLCS scores and for 16% of scores on children’s total life stressors. The total amount of missing data across the entire dataset was approximately 7%, so analyses for missing data and imputation were deemed necessary.

Independent t-tests showed that families who returned for the second session did not differ from those who did not did not return on their CLCS scores, *t*(86) = −40, *p* = 0.693. Missing data analyses were conducted for all variables used in the first regression (CLCS tablet scores, children’s total life stressors, family demographics, total parental distress, and total parental ACEs) using Little’s Missing Completely at Random (MCAR) test. Based on Little’s MCAR test, data were missing completely at random, *χ*^2^ = 25.16, *df* = 25, *p* = 0.454, and missing data was imputed across 20 datasets in SPSS Version 24. All regressions were reconducted with imputed data, and results were pooled across all 20 sets and compared to results from the raw, non-imputed data [68,69]. Results from the pooled datasets did not differ in significance from results using the raw data, and therefore, results using the raw data set are reported.

## 3. Results

Participants were 99 biological parents recruited from an urban emergency homeless shelter during the summers of 2016 and 2017 (92% mothers; *M* age = 30.11 years, *SD* = 7.45, range = 18–62; 72% African-American, 13% Caucasian, 7% American-Indian, 5% biracial/multiracial, 1% other, and 2% not reported) and their three to six-year-old children (52% female; *M* age = 5.11 years, *SD* = 0.83, range = 3.36–6.98, ethnicity = 67% African-American, 5% Caucasian, 6% American-Indian, 20% biracial/multiracial, and 2% not reported).

Bivariate correlations are shown in Table 1. Higher CLCS scores were significantly correlated with higher scores on the measure of children’s total life stressors (*r* = 0.44, *p* < 0.001). Higher CLCS scores were also significantly associated with higher parental ACEs (*r* = 0.29 *p* = 0.013), and independent samples t-tests revealed significant differences between CLCS scores and the dichotomous ACE variable such that scores were significantly lower when parents had an ACE score of three or fewer (M = 29.80, SD = 22.96) compared to when parents had an ACE score greater than or equal to four (M = 41.86, SD = 28.35; t (69.34) = −2.00, *p* = 0.049). CLCS scores were not significantly correlated with parental total parental distress on the K-6 (*r* = −13, *p* = 0.235), and independent samples t-tests revealed no significant differences between CLCS scores and the K-6 cutoff for psychological distress (t(86) = −18, *p* = 0.856). Moreover, CLCS scores were not significantly correlated with child age, and independent samples t-tests revealed no significant differences between CLCS scores and child sex or between CLCS scores and parent education. Scores on the paper-pencil CLCS were strongly related to scores on the tablet version over a two-week interval (*r* = 0.78, *p* < 0.001). Higher paper-pencil CLCS scores were moderately correlated with higher numbers of children’s total life stressors (*r* = 0.62, *p* < 0.001). A paired sample t-test for the subset of the sample who completed the tablet and paper-pencil versions of the CLCS indicated that scores were significantly higher on the paper version (*M* = 46.86, *SD* = 25.34) than the tablet version (*M* = 34.82, *SD* = 25.51; *t*(33) = −4.20, *p* < 0.001). However, Fisher’s “r to z” test indicated that, for this subset of participants, the correlation between children’s total life stressors and the tablet version of the measure did not significantly differ from the correlation between children’s total life stressors and the paper-pencil version (*z* = −1.09, *p* = 0.258). 

Results from the model comparison indicated that parent-reported children’s total life stressors predicted CLCS scores above and beyond parental ACEs, parental distress, and family demographic variables. This model accounted for approximately 27% of the variance (R^2^) in CLCS scores (*p* = 0.001), indicating that parent-reported children’s total life stressors explained about 15% of the unique variance (ΔR^2^) in CLCS scores. In this model, no other predictors significantly predicted CLCS scores only children’s total life stressors (ß = 0.45, *p* = 0.001; see Table 2). Follow-up analyses examining potential moderation by these variables as well as categorical measures of parental distress and parental ACEs showed no significant interactions. 

## 4. Discussion

The current study introduces a new measure of lifetime adversity, the Child Life Challenges Scale, and examines its validity in a sample of families residing in emergency housing. Parents’ ratings on the CLCS were expected to be associated with their reports of their children’s total life stressors, and results indicated a moderate association. Results suggest that parents consider multiple life stressors when marking their ratings on the CLCS. Results also suggest, however, that, in marking their ratings, parents may be drawing on information from other sources as well. Thus, current results indicate that the CLCS shows promise as a measure of lifetime adversity that integrates different aspects of life challenges, including the number and severity, but further research is needed to better understand parental reports on this measure. 

Consistent with the first and second hypotheses, parents’ ratings on the CLCS were related to children’s total life stressors as reported on the LTE-C, and this pattern was true for both the tablet and paper-pencil versions of the measure. Strong correlations between the tablet and paper-pencil versions of the CLCS, as well as comparable correlations with children’s total life stressors obtained with both versions of this measure, support the possibility of using the CLCS in multiple formats. The current study focused mainly on the electronic version of this measure, which is easy to use in settings where tablets are accessible, such as some primary care clinics. The paper-pencil version of this measure may be well suited to use in settings without access to the internet or tablets. For example, a paper-pencil version of this measure would likely be more suitable for lower-income contexts or following adversities such as natural disasters where conditions become impoverished or deprived of resources. The CLCS is therefore not only easy and quick to administer but also can be used in multiple formats and settings. 

Of note, the paper-pencil mean was approximately 12 points higher than the tablet mean, and this difference was significant. The two administration methods may engender different response sets given a blank line to mark versus a tablet slider set that starts in the middle. While these results indicate that participants scored higher on the paper version than the tablet version on average, there was no significant difference in the relationship of scores using these methods, with a theoretical relevant external variable corroborating that scores on both measures were equally valid (i.e., both showed convergent validity). Although correlational patterns for both versions of the measure were not significantly different, future studies should continue to explore how and why the two formats of the measure may differ, especially in other populations and larger samples. If consistent differences are found in the means for different administration methods, then it would be important to know why and to develop separate norms for each method. 

Parents’ reports of their children’s total life stressors predicted parent ratings on the CLCS above and beyond the other variables examined that may influence parental report, consistent with the third hypothesis. Notably, the CLCS and children’s total life stressors were only moderately correlated and the regression accounting for family demographics, parental distress, and parental ACEs in addition to children’s total life stressors only accounted for 27% of the variance in CLCS scores. These results suggest that, in addition to the number of children’s total life stressors, there may be other factors that parents are considering in marking their ratings on the CLCS. Although beyond the scope of the current study, future research is needed to understand what those factors may be. Future work could include cognitive interviews with parents to better understand how they are making their ratings [70] as well as studying other factors that may be related to parental report on the CLCS. For example, through their research on the concordance between parental- and child-report of trauma and trauma-related symptoms, Stover and colleagues concluded that parental understanding of trauma influenced their reports [71]. More information is needed on parents’ views on the impact of adverse events as well as on how this understanding relates to their ratings of their children’s life challenges on the CLCS, and exploration of additional influences on parental ratings is key to further validating this measure. 

The interaction analyses conducted to address the fourth aim of the study indicated that parental history of adversity, parental distress, and family demographics did not moderate the relationship between children’s total life stressors and CLCS scores. The CLCS is therefore likely to be appropriate for diverse parents, including those who are distressed or traumatized. These nonsignificant interactions also strengthen the feasibility of the instrument to administer. Because the CLCS appears to be a valid instrument for most parents, regardless of parental ACEs, parental distress, or family demographics, the measure has the potential to be administered without the necessity of collecting additional information about parental functioning to ensure validity. It is important to note that these results may be the result of limited power due to the small sample size and, therefore, should be interpreted with caution and replicated using a larger sample.

It is noteworthy that this pattern of result appears to be inconsistent with previous research, suggesting that parents’ own childhood adversities affect how they report their children’s experiences of adversity [59]. The current study, however, did not consider shared histories of adversity, as Cohodes and her colleagues did, but rather studied parents’ childhood adversity more broadly. Parents’ childhood adversities as reported on the ACEs may not be the same adversities that their children are currently experiencing, as reported on the LTE-C and the CLCS. Future research looking more specifically at the types of adversities that both parents and children experience is needed. Further, this body of research should consider how this “match” or lack thereof relates to CLCS scores in order to confirm whether the reporting biases noted by Cohodes and colleagues may play a role in parental CLCS reports. Additionally, while previous research has documented adult inconsistencies in retrospective reporting due to high levels of distress [60], these patterns were not observed in parents’ responses to the CLCS. Past research on this topic has been conducted using self-reports of retrospective adversity rather than parental reports of children’s retrospective adversity, and it is therefore possible that while self-report may be affected by distress, reporting on the adversity of others, including children, is not affected in this same way. 

### 4.1. Strengths, Limitations, and Future Directions

This study introduces a quick, low burden method for assessing cumulative life stress among children. This instrument reduces caregiver burden and potentially unnecessary detail that may be distressing or difficult to report because parents respond to one general question about the stressors that have accumulated in their children’s lives to date rather completing a lengthier or detailed checklist of their children’s adversity exposure. The CLCS therefore provides an innovative method through which adversity can be assessed while directly addressing the weaknesses of the cumulative risk approach [20,21] and the concerns that both providers and respondents have put forth around the topic of asking about past traumas and adversities [38,50]. 

Despite these strengths, this study also had several limitations. First, for participants missing data, parental ACEs, parental distress, and children’s total life stressors may be underestimated. The current study is also limited by small sample size, which may have affected moderation analyses as well as a modest level of attrition. Data were drawn from a single shelter, therefore limiting sample characteristics to very low-income families from this shelter. Future work is needed to examine the CLCS in other high-risk populations (e.g., low-income but housed, clinical populations, etc.) and to investigate whether the CLCS operates similarly at lower levels of risk. Until this instrument is examined in other populations as well as larger and more diverse samples, its generalizability is limited. More broadly, the CLCS relies on parental reports of children’s retrospective adversity and was validated using parental-self-report measures of parental distress and adversity. All data were therefore collected from the same informant, and parents’ responses on the CLCS and the patterns described above may also be influenced by social desirability such that participants may not have accurately reported on all of the events that they or their children had experienced or may have marked their rating closer to the left side of the scale (“few mildly challenging experiences”) than they felt accurately portrayed their situation. Other unexamined factors, including reporting biases or retrospective reports of children’s life stressors, may have also influenced results. Future longitudinal work examining parents’ reports on the CLCS as well as changes in these scores while concurrently collecting information on children’s life stressors as they occur is needed to further validate this instrument. Future studies should also consider asking parents why they chose to mark or tap the line in a certain location after parents complete the measure. Parents’ answers to this question may help to further clarify the other factors that parents are considering when responding to the CLCS as well as why children’s total life stressors do not more strongly predict CLCS scores. Finally, reliability data could not be calculated due to the single-item nature of this instrument. Future research examining participants’ responses to both versions of this measure concurrently rather than over a 1–2week period would provide additional reliability information on this measure. 

In addition to the future directions described above, future work examining the CLCS with other, lengthier checklists of children’s stressful life events (in addition to the LTE-C) is needed in order to establish how to best utilize the CLCS as a screener in primary care and community settings. By examining how this instrument relates to an objective number of stressors on different instruments and/or to biomarkers of “toxic stress” such as diurnal cortisol, a cutoff CLCS score for screening could possibly be established to alert providers that they need to further assess stress and adversity. Additionally, future work should consider how multiple informants may respond to the CLCS differently as well as the predictive validity of this measure over a child’s lifetime. Future research should also consider developing a youth self-report version of this measure in addition to the adult self-report version that is currently being validated by our research group [72].

### 4.2. Implications and Conclusions

To our knowledge, the CLCS is the first instrument to examine lifetime adversity exposure globally rather than by asking about exposure to discrete events. Although it is not ready for deployment in non-research contexts, this instrument shows promise. CLCS scores are predicted by the objective number of stressful life events children have experienced, yet parents are more easily and quickly able to complete the measure than life stressors checklists. With further validation and clinical studies, the CLCS could be implemented into primary care and community settings, such as pediatric clinics, to provide a quick screening tool to measure children’s lifetime adversity and then to decide, based on the score, whether it would be helpful to probe the history of life experiences further. Additionally, if providers are aware that a child has experienced a high number of total life stressors from medical or other records but note that parents respond to the CLCS with a low score, they could ask parents why they responded in a certain way as a meaningful strategy for learning more about the family. The current study is the first step in validating this instrument for future use, initially as a research tool, and with further promising evidence, as a new low-burden screening tool for practitioners. 

## Figures and Tables

**Table 1:** Bivariate Correlations for 99 Homeless Parents.

Primary Variable	1	2	3	4	5
1. Child Life Challenges Scale (CLCS)—tablet version	--				
2. Children’s total life stressors	0.44 **	--			
3. Total parental ACEs	0.29 *	0.35 **	--		
4. Total parental distress	−0.13	−0.39 **	−0.26 *	--	
5. Child age	−0.04	0.21	0.05	0.07	--

** *p* < 0.01, * *p* < 0.05.

**Table 2:** Regression for CLCS scores (n = 69).

	Child Life Challenges Scale					
	B	SE	*ß*	R^2^	F	∆R^2^
**Step 1**				0.12	1.74	0.12
Total parental ACEs	2.38	1.07	0.27 *			
Total parental distress	−0.84	0.66	−0.16			
Child age	−2.14	4.17	−0.06			
Child sex	1.81	6.66	0.03			
Parent education	0.16	6.84	0.00			
**Step 2**				0.27	3.74 **	0.15 **
Total parental ACEs	1.28	1.04	0.15			
Total parental distress	−0.06	0.65	−0.01			
Child age	−4.29	3.89	−0.13			
Child sex	4.29	6.18	0.08			
Parent education	−2.32	6.34	−0.04			
Children’s total life stressors	3.23	0.93	0.45 **			

** *p* < 0.01, * *p* < 0.05.
